# Biocompatible PEGylated Fe_3_O_4_ Nanoparticles as Photothermal Agents for Near-Infrared Light Modulated Cancer Therapy

**DOI:** 10.3390/ijms151018776

**Published:** 2014-10-17

**Authors:** Gang Yuan, Yongjie Yuan, Kan Xu, Qi Luo

**Affiliations:** Department of Neurosurgery, the First Hospital of Jilin University, Changchun 130021, China; E-Mails: yuangangsjwk@yeah.net (G.Y.); yongjieyuan@yeah.net (Y.Y.)

**Keywords:** magnetic nanoparticles, NIR, phototherapy therapy, biocompatible

## Abstract

In accordance with the World Cancer Report, cancer has become the leading cause of mortality worldwide, and various therapeutic strategies have been developed at the same time. In the present study, biocompatible magnetic nanoparticles were designed and synthesized as high-performance photothermal agents for near-infrared light mediated cancer therapy *in vitro*. Via a facile one-pot solvothermal method, well-defined PEGylated magnetic nanoparticles (PEG–Fe_3_O_4_) were prepared with cheap inhesion as a first step. Due to the successful coating of PEG molecules on the surface of PEG–Fe_3_O_4_, these nanoparticles exhibited excellent dispersibility and dissolvability in physiological condition. Cytotoxicity based on MTT assays indicated these nanoparticles revealed high biocompatibility and low toxicity towards both Hela cells and C6 cells. After near-infrared (NIR) laser irradiation, the viabilities of C6 cells were effectively suppressed when incubated with the NIR laser activated PEG–Fe_3_O_4_. In addition, detailed photothermal anti-cancer efficacy was evaluated via visual microscope images, demonstrating that our PEG–Fe_3_O_4_ were promising for photothermal therapy of cancer cells.

## 1. Introduction

Over the past decade, cancer has become the main cause of the death worldwide, which has overtaken heart-related disease. Various clinical cancer therapies including surgery, radiotherapy, and chemotherapy have been developed to ablate cancer cells. However, the above approaches are usually limited by the risks of killing normal tissues, an increased incidence of tumor metastasis, and destroying the immune system [1,2,3,4]. As a promising alternative to conventional surgery and other therapies, near-infrared (NIR) laser-induced photothermal therapy (PTT) has emerged as an appealing therapeutic strategy for cancer treatment. By using heart generated from absorbed NIR light energy, PTT can kill cancer cells via a localized “burning” manner. As a result, PTT can availably avoid the risks mentioned above with the minimal invasiveness and high selectivity. In addition, this technique also exhibits several other advantages over traditional approaches, such as simpler procedure, fewer complications, and shorter period in hospital [5,6,7,8,9,10,11,12].

Because of the surface plasmon resonance effects, materials used for PTT must hold a perfect optical absorbance in the region of NIR light. Currently, noble metal-based nanostructures, Cu-based semiconductor nanoparticles, carbon-based nanomaterials, as well as organic polymers and assembly have been well prepared as powerful PTT agents to kill cancer cells both *in vitro* and *in vivo* [13,14,15,16,17,18,19,20,21]. Although promising, these agents cannot achieve the essential clinical implementation due to their unknown long-term toxicity. For example, carbon-based nanotubes and graphene are highly stable under various physiological conditions and can induce oxidative stress and pulmonary inflammation [19,21]. Moreover, metallic nanomaterials are poorly metabolized and have potential toxicity against normal tissues and organs [15,18]. To effectively avoid serious adverse effect caused by unknown long-term toxicity and benefit the practical application in clinic, it is highly important to develop PTT agents with admirable biocompatibility and low systemic toxicity.

As an important family of multifunctional materials, magnetic nanoparticles have been adopted in a wide range of bio-related fields such as magnetic resonance imaging (MRI), targeted drug delivery, cancer treatment, as well as biomolecule separation [22,23,24,25,26]. One excellent example is that superparamagnetic nanoparticles based on Fe_3_O_4_ have been approved as high-performance contrast agents in MRI by Food and Drug Administration (FDA), which indicating the long-term safety of these kinds of nanomaterials and their potential usages in biomedicine [27,28,29,30,31]. Recent studies have demonstrated the design and construction of magnetic nanocomposites and their photothermal effect against bacteria and cancer cells [32,33,34]. However, these systems only focused on the final results of PTT instead of detailed studies including systemic cytotoxicity of nanoagents and induced photothermal toxicity under different treatments. More importantly, PTT against C6 cells have not been studied till now, revealing more potential for glioma treatment along with the cerebral tumor operation.

Inspired by these, here we present a novel PTT agent based on PEGylated Fe_3_O_4_ nanoparticles (PEG–Fe_3_O_4_) for *in vitro* cancer treatment of C6 cells. Biocompatible magnetic nanoparticles were synthesized via a facile one-pot solvothermal route at first. PEG molecules coated on the surface of PEG–Fe_3_O_4_ endowed these nanomaterials with great dispersibility and dissolvability in various physiological conditions. Results of MTT assays indicated the high biocompatibility and low systemic cytotoxicity of PEG–Fe_3_O_4_. When incubated with the NIR laser activated PTT agents, the viabilities of C6 cells were suppressed step by step with the increasing of agent concentration and irradiation period. Last but not least, visual microscope images based different staining methods further demonstrated the unexceptionable photothermal anti-cancer efficacy of our PEG–Fe_3_O_4_ upon NIR laser irradiation with a low laser power density and a short irradiation period.

## 2. Results and Discussion

**Figure 1:**
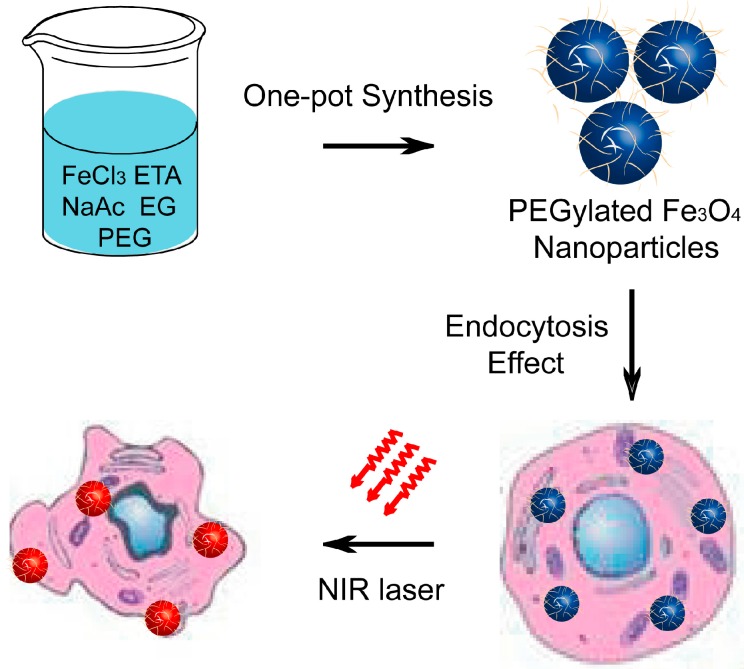
Schematic illustration of the synthesis of PEGylated Fe_3_O_4_ nanoparticles and their application as photothermal agents for cancer therapy under near-infrared (NIR) laser irradiation.

**Figure 2:**
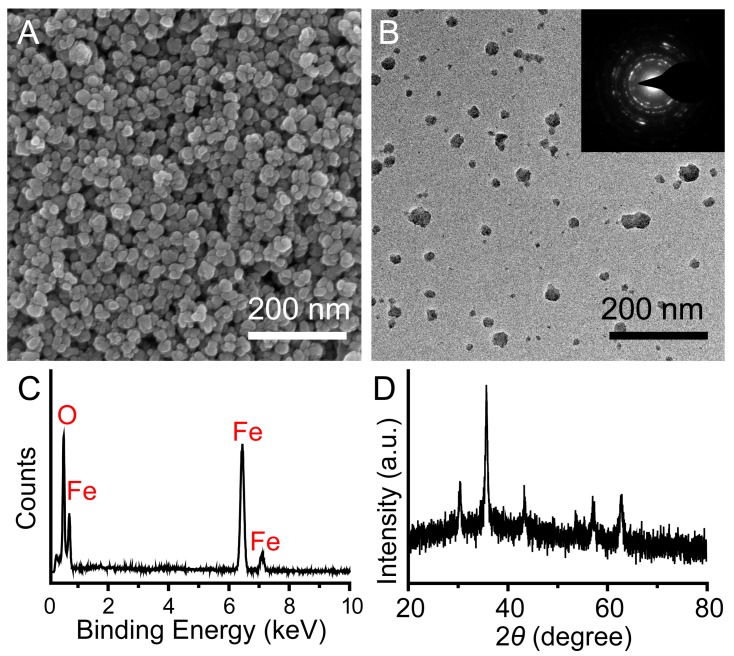
Scanning electron microscope (SEM) image (**A**); transmission electron microscope (TEM) image (**B**); selective area electronic diffraction (SAED) image (inset of (**B**)), energy-dispersive spectroscopy (EDS) spectrum (**C**); and wide-angle X-ray diffraction (XRD) pattern (**D**) of well-prepared PEG–Fe_3_O_4_.

**Figure 3:**
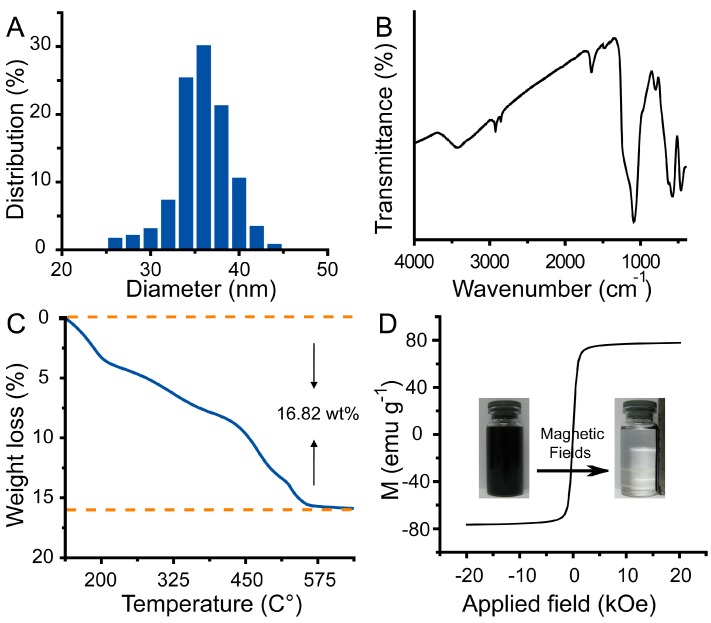
Size distribution (**A**); Fourier transform infrared spectroscopy (FT-IR) spectrum (**B**); Thermalgravimetric analysis (TGA) (**C**); hysteresis loop (**D**); and magnetic separation photo (inset of (**D**)) of PEG–Fe_3_O_4_.

Figure 1 illustrated the synthesis of multifunctional magnetic nanoparticles and their applications as photothermal agents for NIR light modulated cancer treatment *in vitro*. We first prepared well-defined PEG–Fe_3_O_4_ via a one-pot solvothermal route [35,36,37,38]. Scanning electron microscope (SEM) image and transmission electron microscope (TEM) image shown in Figure 2A,B indicated the obtained product was roughly spherical with a mean diameter of 30 nm. Energy-dispersive spectroscopy (EDS) analysis shown in Figure 2C manifested the well distribution of Fe and O in the nanoparticles. Wide-angle X-ray diffraction (XRD) pattern demonstrated that all the diffraction peaks of PEG–Fe_3_O_4_ could be directly indexed to a cubic phase of magnetite, and decoration with PEG molecules could not change the crystal structure of this product (Figure 2D). Otherwise, selective area electronic diffraction (SAED) pattern indicated the polycrystalline nature of these nanoparticles, which held similar results with previous studies (inset of Figure 2B). Dynamic light scattering (DLS) analysis of PEG–Fe_3_O_4_ in 0.9% NaCl solution revealed a much larger average diameter of 37 nm than that of TEM results, foretelling the effective coating of PEG molecules (Figure 3A). To further confirm our above results, FT-IR spectrum of PEG–Fe_3_O_4_ was investigated simultaneously. As shown in Figure 3B, absorption bands around 2942 cm^−1^ associated with the stretching mode of –CH_2_– indicated the existence of PEG in the sample. Determined via thermogravimetric analysis (TGA), the amount of PEG molecules in the sample was approximately 16.82% (Figure 3C). Like some previous studies, PEG molecules acted as both surfactant and coating agents to control the growth of nanoparticles and provide more hydrophilic groups in our system [39]. This successful modification of PEG molecules on surface of PEG–Fe_3_O_4_ could extremely enhance the dissolvability and colloidal stability of these nanoparticles in simulated body fluid (SBF) as expected. Owing to their magnet-assistant separating and targeting features, magnetic nanomaterials have gained significant attention recently. As illustrated in Figure 3D, the magnetization saturation value of PEG–Fe_3_O_4_ was calculated to be 78.53 emu·g^−1^, which was measured via a superconducting quantum interference device (SQUID) magnetometer at room temperature. The photograph inset of Figure 3D presented an admirable dissolution and a visible response of these nanoparticles towards an appropriate magnet. To sum up, our PEG–Fe_3_O_4_ held excellent physicochemical properties and showed more potential for practical applications.

**Figure 4:**
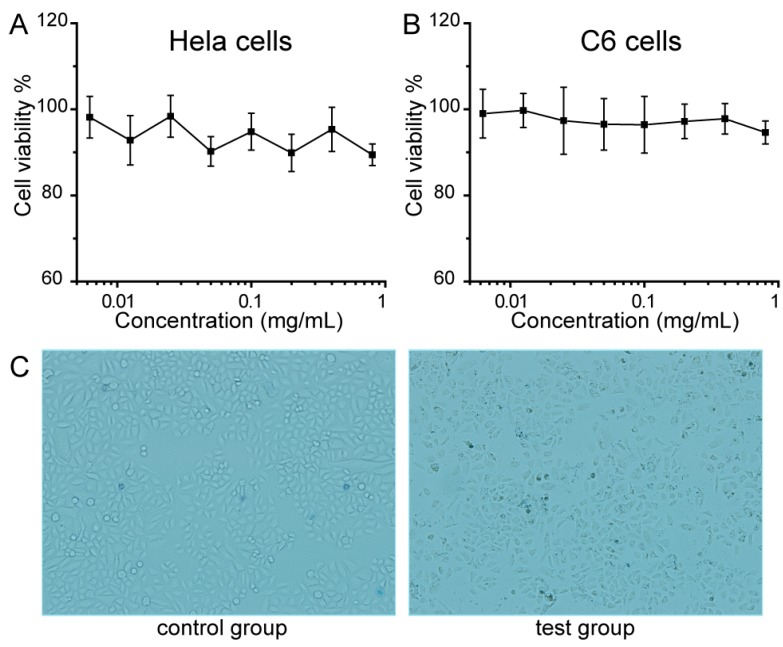
Viability of different cell lines: Hela cells (**A**) and C6 cells (**B**); (**C**) Bright field microscopic images of C6 cells, the control group and test group are incubated without and with PEG–Fe_3_O_4_, respectively. Cell images are observed under a light microscope at 1000× magnification.

Prior to using PEG–Fe_3_O_4_ as a photothermal agent, we investigated their *in vitro* toxicity via the methyl thiazolyl tetrazolium (MTT) assay. This essential factor must be first established and investigated in detail in order to determine the suitability of novel drugs and materials for animal experiments and pre-clinical research. Encouragingly, viabilities of Hela cells and C6 cells were not hindered by PEG–Fe_3_O_4_ up to a concentration of 1 mg·mL^−1^, herein revealing the significantly low cytotoxicity of our magnetic nanoparticles (Figure 4). Moreover, we also investigated the behavior of PEG–Fe_3_O_4_ in living cells via observing the morphological changes. After co-incubation with PEG–Fe_3_O_4_, inverted microscopy images exhibited that all the C6 cells spread and proliferated equally in both the control and test groups. On the basis of the above study of *in vitro* toxicity, it could be inferred that PEG–Fe_3_O_4_ were highly biocompatible and low toxic, and could serve as a safe photothermal agent for cancer therapy.

**Figure 5:**
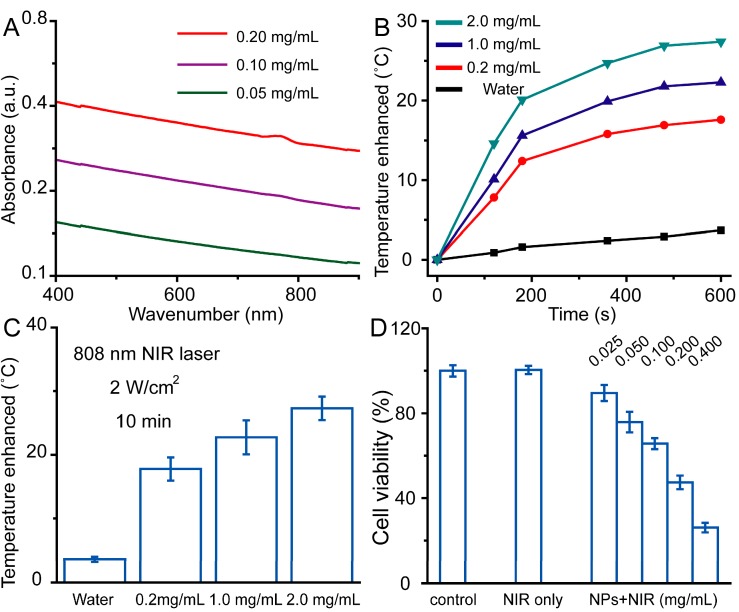
Room temperature UV–vis absorbance spectra of PEG–Fe_3_O_4_ with different concentrations (**A**); Temperature elevation of PEG–Fe_3_O_4_ as a function of time upon exposure to NIR light at 2 W·cm^−2^ (**B**); Statistical analysis of above enhanced temperature based on three independent experiments (**C**); Photothermal effect of PEG–Fe_3_O_4_ on viability of C6 cells with different experimental conditions (**D**).

Due to both the deep penetration of NIR light and low absorption by other healthy tissues, materials with absorbance in the NIR region could be applied as photothermal agents for cancer therapy. With a deep black color, the synthesized PEG–Fe_3_O_4_ exhibited broad absorption ranging from ultraviolet (UV) to NIR wavelengths (Figure 5A). More importantly, the value of absorbance increased with the drug concentrations. To determine the detailed photothermal activity, PEG–Fe_3_O_4_ were dispersed in water at concentrations ranging from 0.2 to 2.0 mg·mL^−1^, and then irradiated with an 808 nm laser at 2 W·cm^−2^ for 10 min. Pure water was selected as a negative control in our design. As shown in Figure 5B, the temperatures of all the PEG–Fe_3_O_4_ samples increased with the irradiation periods, and the temperature increased more rapidly with increasing the concentrations of PEG–Fe_3_O_4_. Three dependent experiments were carried out under the same experimental conditions. Figure 5C revealed that the temperature of solution containing PEG–Fe_3_O_4_ with a concentration of 1.0 mg·mL^−1^ was enhanced by 23.8 °C after NIR laser irradiation. In contrast, the temperature of pure water was only increased by 3.8 °C. Previous studies have demonstrated that cancer cells could be killed after maintenance at 42 °C for half an hour, while this duration also could be shortened to 5 min for temperatures over 50 °C. The normal body temperature of human is between 36 and 37 °C, and hereby cancer cells could be easily heated to over 50 °C within 5 min and be killed efficiently together with the incubation of PEG–Fe_3_O_4_ and NIR laser irradiation. This high photothermal efficiency of PEG–Fe_3_O_4_ prompted us to evaluate their feasibility as photothermal agents to ablate cancer cells. C6 cells were incubated with PEG–Fe_3_O_4_ with different concentrations for half an hour and then exposed to an 808 nm laser at 2 W·cm^−2^ for another 5 min. Figure 5D provided the photothermal cytotoxicity of PEG–Fe_3_O_4_ on C6 cells by using MTT assay. After 24 h of incubation with increasing concentrations of PEG–Fe_3_O_4_, neither the cell viability nor the proliferation of C6 cells was extremely hindered by the presence of these photothermal agents. However, the NIR laser irradiation could not result in the death of cells, indicating the negligible toxicity induced by NIR light with the present irradiation intensity and irradiation period. Moreover, these findings clearly described that the death of C6 cells was mainly caused by the photothermal effect of PEG–Fe_3_O_4_.

**Figure 6:**
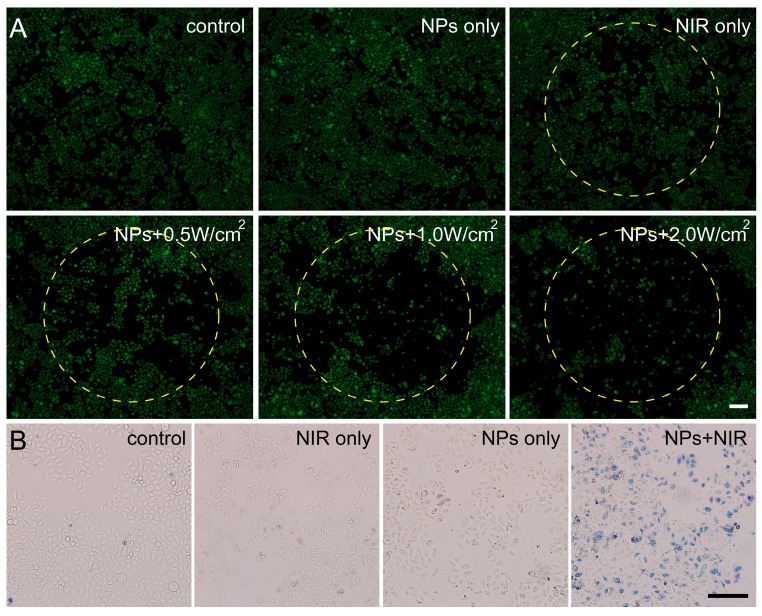
Fluorescence images of calcein AM stained C6 cells (**A**) and optical microscopy images of trypan blue stained C6 cells (**B**) upon different experimental conditions. All scale bars in the pictures represented 200 μm.

To achieve the visible cell viability, fluorescence microscopy was employed in our present study. C6 cells were incubated with PEG–Fe_3_O_4_ for half an hour and then exposed to an 808 nm laser with different intensity for 5 min. After above treatment, C6 cells were stained with calcein AM. As illustrated in Figure 6A, there were no differences between control group, nanoparticle-treated group, and NIR-treated group, revealing the high biocompatibility of PEG–Fe_3_O_4_ and low phototoxicity of our present NIR condition. However, it was found that a significant loss of cell viability happened at 0.5 W·cm^−2^. Otherwise, more killing of C6 cells occurred with increasing the irradiation intensity. White circles were used to denote the areas with NIR laser irradiation. As expected, nearly all the cells were killed in the white circle when C6 cells were directly exposed to the NIR laser at 2 W·cm^−2^ for 5 min. In addition, trypan blue staining shown in Figure 6B also confirmed our above description. Cell deaths occurred only in the group treated with PEG–Fe_3_O_4_ and NIR. Similar with our MTT results and calcein AM-stained images, negligible toxicity happened in the nanoparticle-treated group and NIR-treated group. Thus, it could be deduced that well-prepared PEG–Fe_3_O_4_ could potentially be applied as effective photothermal agents for cancer treatment.

As an important therapeutic strategy, photothermal therapy based on light-absorption nanomaterials has emerged as a powerful technique for cancer treatment [40,41,42]. However, one of the most important issues for the therapy of brain-related diseases is the low permeability to the blood–brain-barrier (BBB) of various agents. Previous studies have proved that nano-based therapeutic agents could cross the BBB with higher amount than some drugs based on small molecules. Moreover, FDA-approved magnetic nanoparticles have been applied as high-resolution contrast agents for MRI including brain-related imaging. More importantly, further modification with targeting molecules could endow these nanoparticles more functionalities in various cancer treatment. Thereby, our well-prepared PEG–Fe_3_O_4_ could accumulate in brain and act as photothermal agents against glioma along with the cerebral tumor operation.

## 3. Experimental Section

### 3.1. Chemicals and Materials

Ferric chloride hexahydrate (FeCl_3_·6H_2_O), anhydrous sodium acetate (NaOAc), polyethylene glycolethylene (PEG-2000), glycol (EG), ethanolamine (ETA), and ethanol were purchased from Beijing Chemicals (Beijing, China). Calcein AM was obtained from Sigma–Aldrich (Shanghai, China). All chemical agents were of analytical grade and used directly without further purification.

### 3.2. Preparation of PEGylated Magnetic Nanoparticles

PEGylated Fe_3_O_4_ nanoparticles were fabricated via one-pot solvothermal method. FeCl_3_·6H_2_O (1.5 g) was dissolved in 40 mL of solvent containing EG (20 mL) and ETA (20 mL) to form a stable orange solution. NaOAc (4.0 g) and PEG-2000 (2.0 g) were added into the above solution under magnetic stirring. The homogeneous solution was transferred to a Teflon-lined stainless-steel autoclave (50 mL) and sealed to heat at 200 °C. After reaction for 10 h, the autoclave was cooled to ambient temperature naturally. The magnetic nanoparticles were washed with ethanol and deionized water in sequence, and then dried in vacuum at 60 °C overnight.

### 3.3. Measurements and Characterizations

A field emission scanning electron microscope (FE-SEM, S4800, Hitachi, Tokyo, Japan) equipped with an energy-dispersive X-ray spectrum was used to determine the morphology and composition of the PEGylated magnetic nanoparticles. Transmission electron microscope (TEM) measurements were carried out on a JEOL JEM-2010EX transmission electron microscope (JEOL, Akishima-shi, Japan) with a tungsten filament at an accelerating voltage of 200 kV. The crystalline structures of the samples were evaluated via X-ray diffraction (XRD) analysis on a Rigaku-Dmax 2500 diffractometer by using Cuκα radiation (λ = 0.15405 nm). The magnetic properties of samples were collected on a MPM5-XL-5 superconducting quantum interference device magnetometer. Thermogravimetric analyses were carried out on a PerkinElmer Pyris Diamond TG/DTA Analyzer (PerkinElmer, Akron, OH, USA), using an oxidant atmosphere (air) with a heating program consisting of a dynamic segment (5 °C/min). All the experiments were performed at room temperature.

### 3.4. Cell Cultures

C6 cells and Hela cells were supplied by American Type Culture Collection. Both cells were cultured in DMEM containing penicillin (100 U·mL^−1^), streptomycin (100 U·mL^−1^), and 10% fetal bovine serum (FBS) in a humidified incubator at 37 °C and 5% CO_2_. Both cells were harvested by the use of trypsin and were re-suspended in the fresh complete medium before plating.

### 3.5. In Vitro Cytotoxicity Studies

MTT assays were carried out to quantify the cytotoxicity of PEG–Fe_3_O_4_. C6 cells and Hela cells were cultured in 96-well plates with a density of 5 × 10^3^ per well for 12 h. After the attachment of the cells, serial dilutions of different formulations containing PEG–Fe_3_O_4_ were added to the culture medium. At the end of the incubation, above medium was removed, and samples were treated with MTT for another 4 h. Then, DMSO was added to these samples to dissolve the formazan crystals. Bio-Rad model-680 microplate reader (Bio-Rad, Hercules, CA, USA) was used to measure the absorbance at a wavelength of 490 nm. Six replicates were done for each treated group and the percent viability was normalized to the cell viability in the absence of PEG–Fe_3_O_4_.

### 3.6. Enhanced Temperature Measure

Solution containing PEG–Fe_3_O_4_ with different concentrations was exposed to an 808 nm laser upon different treatment periods. The enhanced temperature of various samples was collected along with the irradiation periods. The irradiation intensity was denoted as 2 W cm^−2^. Three dependent experiments were respectively performed to result in the final data.

### 3.7. In Vitro Photothermal Toxicity of PEG–Fe_3_O_4_

C6 cells were plated in 96-well plates with a density of 5 × 10^3^ per well for 12 h. DMEM containing PEG–Fe_3_O_4_ with different concentrations were added into above system. To test the photothermal toxicity of PEG–Fe_3_O_4_, these cells were exposed to an 808 nm laser with an intensity of 2 W·cm^−2^ for 5 min. C6 cells were then incubated at 37 °C with 5% CO_2_ for another 24 h. After treatment, these samples were treated with MTT for 4 h. DMSO was added to these samples to dissolve the formazan crystals. Bio-Rad model-680 microplate reader was used to measure the absorbance at a wavelength of 490 nm. Six replicates were done for each treated group and the percent viability was normalized to the cell viability in the absence of PEG–Fe_3_O_4_ and NIR laser. Without the addition of PEG–Fe_3_O_4_, the irradiation intensity and period of NIR laser were denoted as 2 W·cm^−2^ and 5 min to result in the NIR irradiation group.

### 3.8. Fluorescence Microscopy Analysis

C6 cells with a density of 2 × 10^4^ were plated in a 12-well plate for 6 h to allow the cells to attach. After the cells were washed twice with cool 0.9% NaCl solution, PEG–Fe_3_O_4_ with a concentration of 0.4 mg·mL^−1^ was added to above cell culture medium. Upon different treatment shown in Figure 6A, the cells were washed again with 0.9% NaCl solution several times to remove the remaining particles and then stained with calcein AM to further confirm the visualized viability of C6 cells. The irradiation period was denoted as 5 min. The fluorescence images were collected via Olympus BX-51 optical system (Olympus, Tokyo, Japan). In addition, C6 cells were stained with trypan blue for 15 min, washed with 0.9% NaCl solution, and then imaged using a digital microscope. The NIR laser intensity and irradiation period was denoted as 2 W·cm^−2^ and 5 min, respectively.

### 3.9. Statistical Analysis

All data are expressed in the present manuscript as the mean result ± standard deviation (SD). The statistical analysis was performed by using Origin 8.0 software (OriginLab, Wheeling, IL, USA).

## 4. Conclusions

In summary, we have successfully presented a high-performance photothermal therapeutic agent based on PEGylated magnetic nanomaterials. Our well-designed PEG–Fe_3_O_4_ composed of a magnetic core and PEG molecule shell were constructed via a facile one-pot solvothermal method. Due to the FDA-approved standard of magnetic nanoparticls, photothermal agents based on our PEG–Fe_3_O_4_ could effectively avoid serious adverse effects associated with the long-term retention of foreign materials and unknown long-term toxicity. These nanoparticels exhibited photothermal activity, and could efficiently kill cancer cells. Moreover, PEG–Fe_3_O_4_ could suppress the growth and proliferation of C6 cell without damaging other cells without irradiation treatment. We designed and carried out detailed *in vitro* experiments to support our hypothesis. Last but not least, C6 cells were selected in our present study to investigate the photothermal activity of our nanoagents against glioma. Taking together, these above results exhibited the promising potential of PEG–Fe_3_O_4_ in photothermal killing of cancer cells.
